# A Complex Facial Trauma Case with Multiple Mandibular Fractures and Dentoalveolar Injuries

**DOI:** 10.1155/2015/301013

**Published:** 2015-08-03

**Authors:** Yeliz Guven, Sevgi Zorlu, Abdulkadir Burak Cankaya, Oya Aktoren, Koray Gencay

**Affiliations:** ^1^Department of Pedodontics, Faculty of Dentistry, Istanbul University, Capa, 34093 Istanbul, Turkey; ^2^Department of Pedodontics, Faculty of Dentistry, Aydin University, Sefaköy, 34295 Istanbul, Turkey; ^3^Department of Oral and Maxillofacial Surgery, Faculty of Dentistry, Istanbul University, Capa, 34093 Istanbul, Turkey

## Abstract

The principles of management of mandibular fractures differ in children when compared to adults and depend on the specific age-related status of the growing mandible and the developing dentition. This paper presents a case report with a complex facial trauma affecting the mandibular body and condyle region and dentoalveolar complex. Clinical examination revealed soft tissue injuries, limited mouth opening, lateral deviation of the mandible, an avulsed incisor, a subluxated incisor, and a fractured crown. CBCT examination revealed a nondisplaced fracture and an oblique greenstick fracture of the mandibular body and unilateral fracture of the condyle. Closed reduction technique was chosen to manage fractures of the mandible. Favorable healing outcomes on multiple fractures of the mandible throughout the 6-year follow-up period proved the success of the conservative treatment. This case report is important since it presents a variety of pathological sequelae to trauma within one case.

## 1. Introduction

Less than 15% of all facial fractures take place in pediatric age groups and these occur very rarely (1%) in children under 5 years of age. The incidence rises as children start school and peaks during puberty and adolescence due to increased unsupervised physical activity [1–3]. Mandibular fractures are the most common facial fractures seen in hospitalized children. The reported incidence of mandibular fractures is approximately 20–50% of all childhood facial fractures [4]. The most frequent site of pediatric mandibular fractures is the condylar region, followed by the symphysis/parasymphysis, angle, and body, respectively [5–7].

A thorough clinical examination is important in evaluating a suspected mandibular trauma. A hematoma in the floor of the mouth or a laceration of the gingiva adjacent to the teeth can indicate the presence of fractures in the mandibular symphysis or body regions. Mobility of the fractured segments should also be evaluated by palpation. The condylar region should be carefully inspected for any evidence of fracture, including pain, restricted movement, deviation, crepitus, trismus, and open bite as the patients actively open and close their mouths [8]. The diagnosis should be confirmed by panoramic or posteroanterior mandible radiographs, or if possible by cone beam computed tomography (CBCT) radiographs.

In all types of mandibular fractures, the primary focus of the treatment is the restoration of function while minimizing the side effects on mandibular growth. Particularly in growing children, it should be remembered that the management of injuries to the mandible has significant implications with respect to future craniofacial growth, development, and function [7, 9].

The purpose of this case report is to present the clinical and radiographic evaluation and management of a child who suffered a facial trauma resulting in fractures of the mandibular body and condyle, tooth avulsion, and horizontal root fracture. Six-year follow-up results are also presented.

## 2. Case Presentation

**Figure 1:**
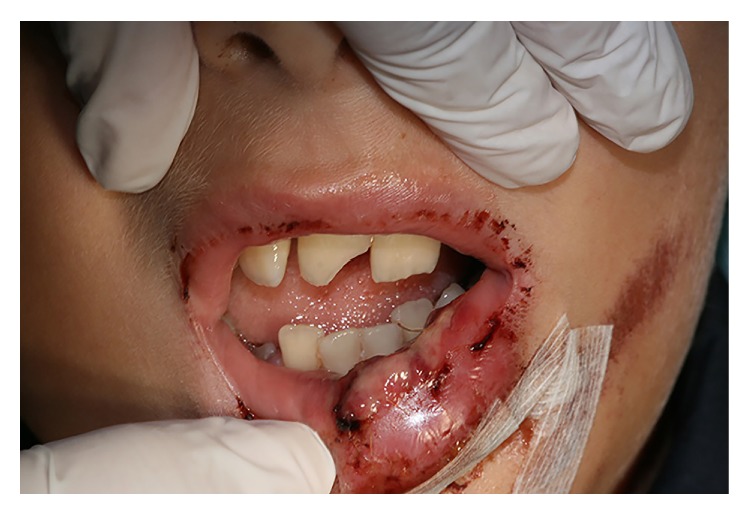
Preoperative view of the limited mouth opening and soft tissue wounds of the case-study patient following a car accident.

**Figure 2:**
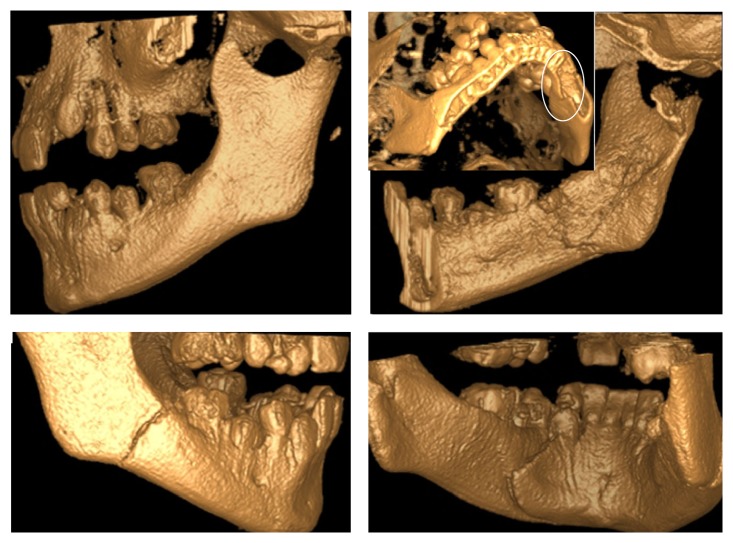
Cone beam CT views showing the fractures in the mandibular body.

**Figure 3:**
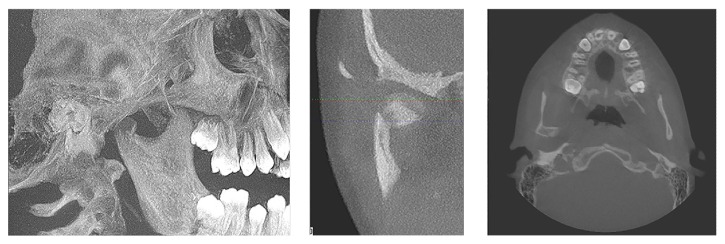
3D, axial, and coronal views of the right condyle, showing the medially displaced subcondylar fracture.

**Figure 4:**
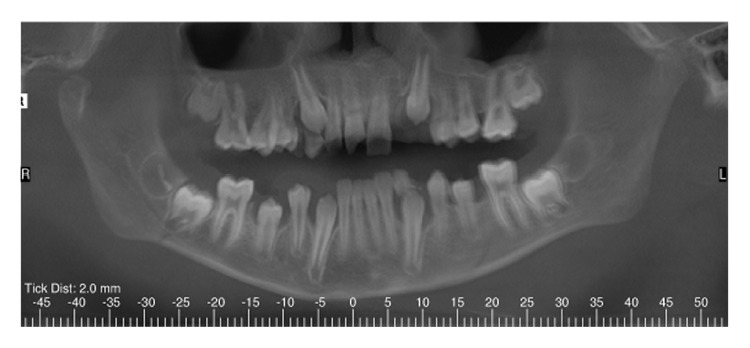
Initial panoramic radiography showing mandibular body fractures, avulsed maxillary left lateral incisor, and maxillary right central incisor with horizontal root fracture.

**Figure 5:**
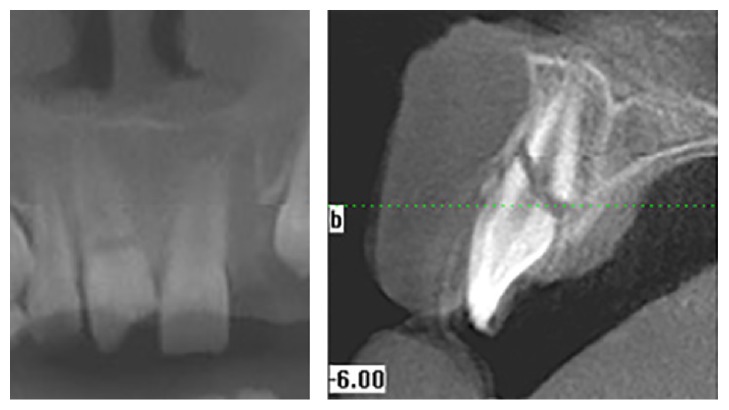
Cross-sectional and coronal view of the horizontal root fracture.

A healthy 11-year-old boy was referred to the clinics of Department of Pediatric Dentistry, following a severe facial trauma as a result of automobile accident. He complained of pain in his jaw and was unable to open his mouth. Initial physical examination showed abrasions and lacerations on the facial skin and the lips (Figure 1). Limited opening of the mouth and lateral deviation of the mandible toward the right side on mouth opening were noted. Intraoral examination revealed a missing maxillary left permanent lateral incisor, an uncomplicated crown fracture of his maxillary right central incisor, and subluxation of his maxillary left central incisor. CBCT examination revealed a nondisplaced mandibular body fracture in the right second molar region, an oblique greenstick fracture on the left lingual side extending between the canine and first molar tooth, and a unilateral medially displaced subcondylar fracture on the right side (Figures 2-3). The vertical height of the ramus was decreased in fractured side. Avulsion of the maxillary left lateral incisor was verified by radiograph and a horizontal root fracture of the maxillary right central incisor was also detected (Figures 4 and 5). The maxillary lateral incisor was lost at the site of the accident.

**Figure 6:**
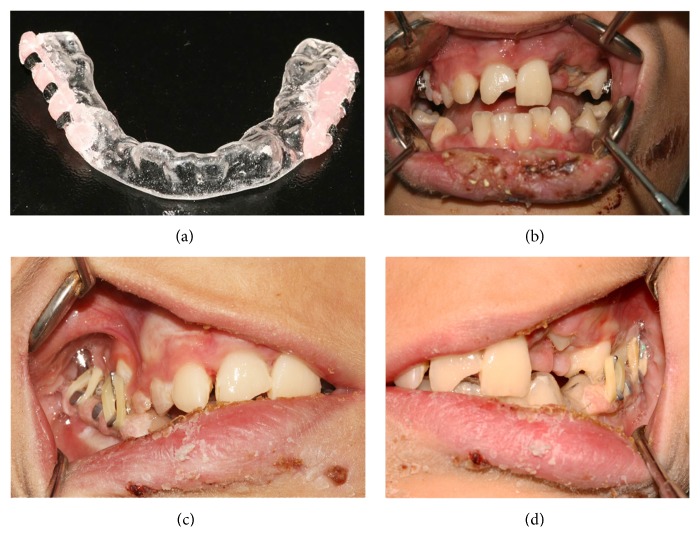
(a) Vacuum formed splint used for functional repositioning. (b) Intraoral photograph showing the brackets attached to the maxillary posterior teeth. (c-d) Orthodontic elastics guide the patient into centric occlusion.

Closed reduction techniques were chosen to manage the mandible fractures. A vacuum formed splint was fitted in the lower arch for functional repositioning of the mandible (Figure 6(a)). Arch bars that were cut to the appropriate size were bonded to vestibuloposterior parts of the splint using acrylic, and brackets were attached to the maxillary posterior teeth (Figure 6(b)). Orthodontic elastics (1/4 inch, medium strength) were used to prevent uncontrolled movements of the jaw within the first week of treatment (Figures 6(c)-6(d)). The splint helped the child avoid doing excessive mouth opening and closing movements. The patient was instructed to wear the splint continuously during 24 h removing it only for eating and cleaning. A soft diet was also recommended. Analgesics and antibiotics were prescribed and the patient was instructed to gargle gently with chlorhexidine mouthwash for a week. One week later, the splint's height was increased to about 3 mm on the fractured side of the condyle and this remained for 5 weeks until a stable occlusion had been achieved. After two months, the right central incisor did not respond to the electric pulp test on clinical examination. Endodontic therapy was performed in the coronal fragment only and mineral trioxide aggregate (MTA) was used for the permanent root canal filling.

**Figure 7:**
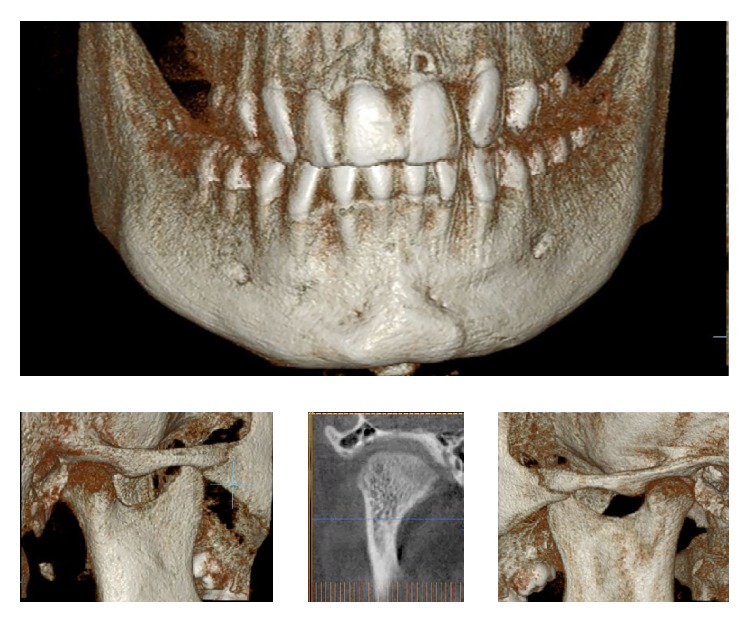
Cone beam CT views of the mandible after 18 months.

**Figure 8:**
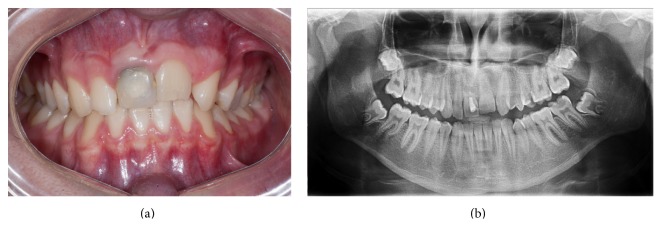
Six-year follow-up findings. (a) Intraoral photograph showing the discoloration of the maxillary right central incisor, infraocclusion of both central incisors, and midline shift. (b) Panoramic radiograph showing internal resorption in the maxillary right central incisor and ankylosis of the left central incisor.

Clinical and radiological examination after 18 months revealed uneventful healing with reduction of the condylar head and remodeling of the condylar process following the conservative treatment and also complete healing of the mandibular body fractures (Figure 7). The patient was not available for the follow-up controls. At the age of 17 years, he visited our clinics again with a complaint of discoloration on his maxillary right central incisor. Clinically, the maxillary central incisors were in infraposition, being more prominent in the left incisor than the right incisor (Figure 8(a)). Radiographic examination showed internal resorption on the maxillary right central incisor and ankylosis on the maxillary left central incisor (Figure 8(b)). The height of the ramus in the fractured side was similar to that of unfractured side and the mandible showed no deviation during mouth opening and closing movements. The ongoing treatment plan for the patient involves prosthetic rehabilitation of the maxillary incisors subsequent to extraction of the maxillary right central incisor and implant placement.

## 3. Discussion

The mandible is divided into specific anatomic areas (symphysis, body, angle, ramus, coronoid, and condyle), and a fracture of the mandible is often described by the location of the fracture in one or several of these areas. They may also be classified as greenstick (nondisplaced), displaced, or comminuted. Another classification is based on location and configuration and described as favorable or unfavorable [10, 11]. Condylar fractures can be classified as intracapsular (condyle head) and extracapsular (condyle neck and subcondylar) based on the fracture position; “nondisplaced, deviated, displaced, and dislocated” according to the location of the condylar head and articular fossa; or “medial, lateral, no overlap, or fissure” according to the extent of dislocation [6, 12]. In the present case report, a complex fracture on multiple sites involving mandibular body fractures without displacement (greenstick fracture) on both sides of the mandible and a unilateral medially displaced subcondylar fracture on the right condyle were observed.

There are two principal therapeutic approaches to these fractures: conservative and surgical. Treatment of mandibular fractures in children depends on the fracture type and the stage of skeletal and dental development. The main goal of treatment is to restore the underlying bony architecture to its preinjury position as noninvasively as possible with minimal residual esthetic and functional impairments. Active mandibular growth centers and permanent tooth buds located in close proximity to the mandibular and mental nerves should be considered when choosing the mode of treatment [1, 8]. Greenstick fractures of the angle, body, or parasymphyseal regions of the mandible as in this case are common in childhood and they had a favorable outcome due to the periosteal sleeve which enables rapid union of the fractured segments. The disruption of the soft tissue and periosteal envelope of the mandible may have deleterious effects on growth. Accordingly, mandibular fracture without displacement and malocclusion are managed by close observation, a soft diet, avoidance of physical activities, and analgesics [8, 13]. In this case, the fractures on the body of the mandible were greenstick type and managed by closed reduction. The mandibular condyle is one of the major growth sites, with a great capacity to adapt to changes in its relationship to its surrounding structure during development. This remodeling capacity of the condylar process enables regeneration of the fractured condyle to approximately its original size in most cases if properly managed [14, 15]. In adult patients, the fractures have a lower potential for remodeling, and condylar fractures with dislocation have less predictability in relation to adaptation and bone remodeling. Thus, the need for surgical reduction of the fracture to replace the condyle within the articular fossa is greater after the end of the growth phase. It is important to note that usually the surgical reduction of condylar fractures is a delicate procedure due to the presence of several anatomical structures in the region and difficulty of manipulation of fractured segments especially when the condyle is displaced medially [16]. The lateral pterygoid muscle which is inserted into the pterygoid fovea under the condylar process of the mandible pushes the fragments of the mandible anteriorly and medially during mouth-opening movements. When an occlusal splint is used, the mouth stays in a slightly open position, thus preventing the contraction forces of the lateral pterygoid muscle and allowing condylar remodeling [17]. In the present case, the patient used an occlusal splint for 5 weeks in order to maintain the remodeling of the fractured condyle. Boffano et al. investigated the outcomes of the conservative treatment of unilateral displaced condylar fractures in a series of children with mixed dentition and their treatment protocol included the splints with increased posterior height on the fractured side as in the present study. They emphasized that the remodeling of the fractured condyle was guided by the increased posterior height of the splint which was progressively remodeled to maintain a good and stable occlusal plane [18]. Farronato et al. explained the reason for gradual increase of the splint's height on the side of the fracture as obtaining a fulcrum for remodeling of the condyle [14].

Plain radiographs in children can be inadequate for assessment of mandibular fractures, due to the greenstick nature of the fracture and the unerupted tooth buds obscuring the fractures [8]. Particularly in cases of intracapsular or sagittal fractures of the condyles, a CT scan is essential in order to increase the diagnostic accuracy as it allows a detailed examination of the affected side in different anatomic planes. In the present case, the patient had a complex fracture of the mandible involving the mandibular body and condyle region, which could not be assessed easily and effectively on conventional radiographs [16, 19]. Therefore, a CBCT scan, which provides a lower radiation dose in comparison with the conventional CT, was performed.

If fractures of the mandibular condyle in children are left untreated or are not properly managed, some complications may arise, including facial asymmetry, malocclusion, disturbance of mandibular movement and occlusal condition, and ankylosis [16, 20, 21]. Ankylosis has a greater chance of development in children; this is attributed to the high condylar vascularization and greater bone-healing capacity in the first years of life, which offer a high potential for remodeling in growing patients [22]. In this case report, none of these complications was observed at the 6-year follow-up. Radiographic examination revealed that the remodeling of the condyle was very good and that the function was within normal ranges.

Malocclusion following closed or open reduction of mandibular condylar fractures is a typical clinical finding due to the loss of vertical height of the ramus. This loss in height frequently results in deviation of the mandible to the affected side in cases with unilateral displaced condylar fractures or anterior open bite in cases of bilateral condylar fractures [23]. In the present case, the vertical height of the ramus was decreased due to the unilateral fracture in the subcondylar region. At the last follow-up, the height of the ramus in the fractured side was similar to that of unfractured side and the mandible showed no deviation during mouth opening and closing movements.

## 4. Conclusion

In the present case report, conservative treatment of mandibular body fractures and a unilateral displaced condylar fracture in the child showed satisfactory functional outcomes at a 6-year follow-up. An appropriate splint guided the correct remodeling of the condyles and allowed restoration of the normal shape and height of the fractured process. Although nonsurgical management should be considered as primary preferred method in children with mandibular fractures, each case should be evaluated individually.
